# Correlation between inflammation, vascularization, adipocyte percentage and exocrine cell percentage at the resection margin and the postoperative pancreatic fistula rate after distal pancreatectomy

**DOI:** 10.1007/s00423-026-04010-9

**Published:** 2026-03-09

**Authors:** Viola Palecek, Tutku Tüfekçi, Phillip Gärtner, Alexander Muckenhuber, Carsten Jäger, Rüdiger Göß, Ilaria Pergolini, Carmen Mota Reyes, Sergey Tokalov, Okan Safak, Mert Erkan, Helmut Friess, Rouzanna Istvanffy, Güralp Onur Ceyhan, Ihsan Ekin Demir, Elke Demir

**Affiliations:** 1 Department of Surgery, Klinikum rechts der Isar, School of Medicine, TUM University Hospital, Munich, Germany; 2 German Cancer Consortium (DKTK), Partner Site Munich, Munich, Germany; 3 Department of Surgery, Koc University School of Medicine, Istanbul, Turkey; 4 Institute of Pathology, Technical University of Munich, Munich, Germany; 5 Department of General Surgery, HPB-Unit, School of Medicine, Acibadem Mehmet Ali Aydinlar University, Istanbul, Turkey; 6 Department of Surgery, Klinikum rechts der Isar, TUM University Hospital, Ismaninger Str. 22, D-81675 München, Germany

**Keywords:** Pancreas, Surgery, Fistula, Stump, Leukocytes, Macrophages, Vascularization, Adipocytes, Exocrine cells

## Abstract

**Background:**

Postoperative pancreatic fistula (POPF) remains the most common and impactful complication after distal (left) pancreatectomy (DP). While previous studies have identified histological features such as acinar cell content and fibrosis as risk factors for POPF after pancreatoduodenectomy (PD), their relevance in DP remains unclear. The aim of this study was to investigate whether histopathological features at the pancreatic transection margin are associated with clinically relevant POPF after DP.

**Methods:**

This retrospective pilot study included 51 patients who underwent DP between 2019 and 2022 at the Department of Surgery, Klinikum rechts der Isar, Technical University of Munich. Immunohistochemical staining of pancreatic resection (transection) margin tissue was performed for leukocytes (CD45), M1 macrophages (CD68), endothelial cells (CD31), exocrine cells (PanCK), and adipocytes. Quantitative analysis was conducted using the QuPath digital pathology platform, and associations between these histological features and clinically relevant POPF were assessed.

**Results:**

CR-POPF occurred in 21 of 51 patients (41.2%). No significant differences were observed in the area fractions of CD45 (*p* = 0.139), CD68 (*p* = 0.318), CD31 (*p* = 0.476), PanCK (*p* = 0.656), or adipocyte content (*p* = 0.398) between the POPF and No POPF groups. Histological features at the pancreatic resection margin did not correlate with the development of POPF.

**Conclusion:**

Unlike in PD, histological composition of the pancreatic stump—specifically inflammation, vascularity, adipocyte content, and exocrine tissue—does not appear to predict POPF after DP. These findings suggest that technical, mechanical, and postoperative management factors may play a more dominant role in fistula formation in this context. Further prospective, multicenter studies are needed to validate these observations and guide risk stratification strategies.

## Introduction

Distal pancreatectomy (DP) is consistently associated with a postoperative pancreatic fistula (POPF) rate ranging from 15% to 35%, with clinically relevant fistulas accounting for the majority of these cases [1–4]. It is considered the “Achilles’ heel” of pancreatic surgery due to its representation of a major source of morbidity. Once POPF develops, it not only prolongs hospital stay but also exposes patients to serious complications such as intra-abdominal abscess, sepsis, hemorrhage, or the need for interventional drainage or reoperation, all of which increase healthcare costs and postoperative burden [2, 5]. Previous studies identified multiple factors that have been associated with the development of POPF after DP. Patient-related factors such as high body mass index (BMI), hypoalbuminemia, younger age; [1, 2] surgery-related factors such as prolonged operation time, increased blood transfusion, open approach, vascular resection; and morphological factors such as underlying pathology, pancreatic duct diameter, pancreatic neck thickness and pancreas consistency have been shown to be in relation to POPF [1, 2, 4, 6]. 

Although previous studies have reported a higher POPF rate after DP than pancreatoduodenectomy (PD), few have sought to explain the reason for this difference. Previous studies have predominantly focused on patients undergoing PD with pancreato-enteric anastomosis. Pancreatic texture—specifically the combination of low fibrosis and high fat content—is a well-established risk factor for POPF after PD. However, whether these histological features carry the same predictive value in DP remains unclear. Neither pancreatic steatosis nor the degree of fibrosis was significantly associated with POPF following DP, in contrast to PD where fibrosis showed a clear protective effect [7]. This discrepancy suggests that tissue characteristics at the transection margin may influence fistula formation differently depending on the type of resection.

Unlike pancreatoduodenectomy, which requires pancreatoenteric anastomosis healing, distal pancreatectomy results in a sealed pancreatic stump without mucosal adaptation. Consequently, the mechanisms driving POPF development differ fundamentally between these procedures. While anastomotic integrity and gland texture dominate POPF pathophysiology after PD, stump closure technique, ductal sealing, and local mechanical stress are likely more relevant after DP. Therefore, histological risk factors validated in PD cannot be assumed to have equivalent predictive value in DP, underscoring the need for DP-specific investigations.

Our study aimed to investigate whether histopathological features at the pancreatic resection margin—specifically vascularization, inflammation, adipocyte content, and exocrine cell proportion—are associated with clinically relevant POPF (CR-POPF) after DP. Using blinded, quantitative immunohistochemical analysis, we sought to clarify the biological relevance of these factors in fistula development.

## Materials and methods

### Study design

A retrospective analysis was conducted at the Department of Surgery, Klinikum rechts der Isar, Technical University of Munich, Germany, between February 2019 and August 2022. All consecutive adult patients undergoing elective distal (left) pancreatectomy with pancreatic transection and stump closure were screened for inclusion. Exclusion criteria were insufficient tissue quality for histological analysis and deviation from standardized stump closure techniques.

The primary objective of this study was to investigate whether the occurrence of a POPF correlates with vascularization, inflammation, the adipocyte percentage, and the exocrine cell percentage at the parenchymal resection margin of the pancreatic tissue. The outcome variables were the relative areas of CD45+, CD68+, CD31+, and PanCK+positive cells, as well as adipocytes, measured as a proportion of the total section area of the pancreatic transection margin.

A total 57 patients’ data who underwent DP were retrieved from the digitalized patient records in the hospital’s SAP system retrospectively. Five patients were excluded due to insufficient tissue quality for analysis. One patient was excluded due to not meeting the inclusion criteria related to the surgical technique.

Ethical approval was obtained from the Ethics Committee of the Technical University of Munich (Reference number: 30–17s).

## Surgical technique and center expertise

All procedures were performed at a certified high-volume pancreatic surgery center by four board-certified surgeons with extensive experience (minimum of 150 pancreatic resection as main surgeon previously) in pancreatic resections. Distal pancreatectomy was carried out using a standardized surgical approach, with pancreatic transection at the body or neck level based on tumor location. Stump closure was performed using either stapler closure (in cases with a rather thin tissue thickness at the pancreatic neck) or hand-sewn technique (in cases with a rather thick pancreas, when a stapler failure can be expected) according to intraoperative assessment. Postoperative management followed institutional enhanced recovery protocols, including routine drain placement and standardized monitoring of drain amylase levels.

## POPF definition (per ISGPS)

The study utilized the updated 2017 ISGPS definition and grading of POPF [8]. Grade B POPF was defined as a fistula requiring a change in postoperative management, including prolonged drainage (> 21 days), pharmacological treatment, nutritional support, or percutaneous interventions, without organ failure. Grade C POPF was defined by the presence of organ failure, reoperation, or death.

## Immunohistochemistry and QuPath analyses

In cooperation with the pathological institute of the Klinikum rechts der Isar, the tissue of the pancreatic resection margin was formalin-fixed, and paraffin embedded. To prepare the tissue sections for evaluation, the 4-µm-thick sections were initially deparaffinized using Roticlear^®^ xylene substitute for three cycles of 10 min each. Subsequently, rehydration was carried out using a descending series of ethanol. The specimens were then washed with distilled water for two cycles of 5 min each, followed by a five-minute wash in tris-buffered saline solution with Tween20 (TBST buffer).

The sections were boiled at 600 W microwave for three minutes and then at a temperature of 90 °C for ten minutes in citrate buffer (pH 6.0). After boiling, the sections were allowed to cool for 20 min at room temperature and then washed for five minutes in TBST buffer. Subsequently, the tissue samples were incubated in a humid chamber with 0.5% Triton X-100 phosphate-buffered saline (PBS) and 3% hydrogen peroxide for ten minutes each, with a five-minute TBST buffer wash in between. Following the hydrogen peroxide incubation, a five-minute wash with distilled water was performed. To inhibit other antigen proteins, the sections were treated with “Protein Block Serum-Free Ready-To-Use” from DAKO and stored at room temperature in a humid chamber for ten minutes. The specimens were then deparaffinized, and antigen retrieval was performed using the appropriate instrument. Finally, all slides were incubated with the primary antibody to facilitate specific cell staining. Four distinct immunohistochemical staining were performed for each patient.

**Fig. 1:**
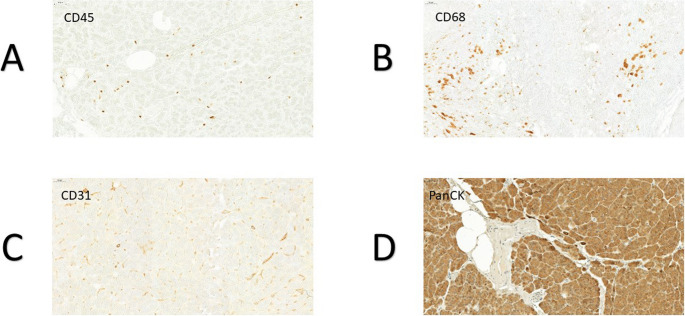
The figures show the four different immunhistochemical stainings of the pancreatic tissue at the resection margin. **A**: CD45-staining. **B**: CD68-staining. **C**: CD31-staining **D**: PanCK-staining

**Fig. 2:**
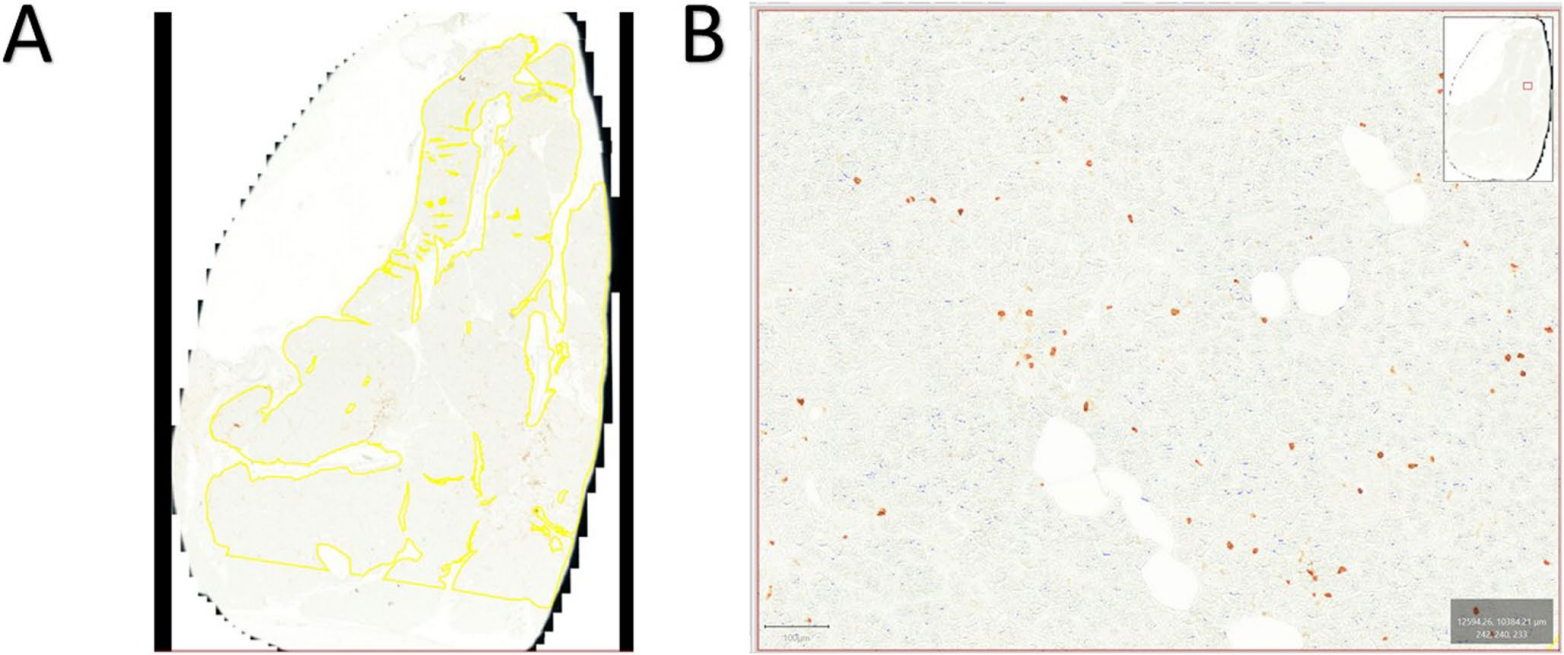
Analysis method using QuPath **A**: Representative tissue area marked on a slide stained (CD45) . **B**: Illustration of the detection of CD45-positive cells

CD45 staining was performed using CD45 leukocyte common antigen (LCA) as the primary antibody (Clone: 30-F11, Rat anti-Mouse, Dilution: 1:20, X minutes at X temperature, Supplier: BD Pharmingen) to detect leukocytes [9]. CD68 staining was performed using CD68-Antibody as the primary antibody (Clone: FA-11, Rat anti-Mouse, Dilution: 1:50, X minutes at X temperature, Supplier: AbD Serotec) to detect macrophages [10]. CD31 (PECAM-1) was stained using CD31-Antibody as the primary antibody (Clone: 1A10, Mouse anti-Human, Dilution: 1:100, X minutes at X temperature, Supplier: Zymed) to detect vascularization [11]. PanCK staining for visualizing epithelial tissue such as acinar and ductal cells was performed using pan-Cytokeratin IHC Antibody as the primary antibody (Clone: Mouse Monoclonal: IML-91, Dilution: X, X minutes at X temperature, Supplier: IHC World) [12]. (Fig. 1). Adipocytes were recognized based on their typical morphology and lipid content (Fig. 2).

Following the incubation period, the tissue sections underwent an additional wash with TBST buffer for three cycles of 10 min each. Subsequently, the sections were subjected to staining using the diaminobenzidine chromogen solution (DAB) under a microscope. The exposure time for DAB was optimized and set at 15 s per slide to ensure optimal staining. After staining, the sections were washed with distilled water for five minutes, and a counterstaining step using hematoxylin was performed for seven seconds per slide. To complete the process, dehydration was carried out using an ascending alcohol series. The sections were then cleaned with Roticlear^®^ for three cycles of 5 min each, and Menzel cover glasses (24 × 32 mm) were firmly affixed onto the specimens using “Vecta Mount permanent Mounting Medium.” Fig. 1 displays the results of the four different immunohistochemical staining of the pancreatic tissue at the resection margin.

After immunohistochemical staining, the specifically stained sections were digitized using a brightfield imaging function of the Zeiss Axio Scan.Z1 Slide Scanner. The resulting whole slide scans were analyzed using the QuPath^®^ image analysis platform (QuPath software version 3.0, Queens’s University of Belfast, Belfast, Nordirland, https://qupath.github.io/) [13]. To measure the area of specifically stained cells within the “region of interest (ROI)”, QuPath’s “positive pixel count” function was applied. To receive a representative positive pixel area to be determined via this function, the DAB threshold must be adjusted, according to the color intensity of the cells to be measured. To ensure comparability between the slides, all analyses use a default down-sample factor of 2.0 and Gaussian sigma of 0.5 μm. These values affect the measurement precision but are limited by the computer’s processing power. The analysis calculates the percentage of specifically stained cells relative to the total pancreatic transection section area (Positive pixel count/total ROI area). Additionally, fat cell area were measured in all stained slides for each patient to determine an average fat cell percentage (Adipocytes area/total ROI area). During the measurements, the examiner was blinded related to the patient’s outcome concerning the development of a POPF. The collected data was separated into two groups (POPF and No POPF) for each staining and fat cell area separately. This allows for an assessment of how frequently each examined cell type appears at the pancreatic resection margin in relation to the development of a POPF.

### Statistical analysis

Data collection, statistical analysis and graph creation were completed GraphPad Prism 9 (Version 9.4.1, GraphPad Software, San Diego, USA). To identify the most appropriate statistical test, a Shapiro-Wilk test was performed to investigate whether the data sets of the five subgroups follow a normal distribution. Additionally, an F-test was conducted to examine the equality of variances between the two groups for each staining. Depending on distribution of the data, either a T-test for independent samples or a Mann-Whitney-U test was conducted to compare the POPF and the No POPF groups. All results were analyzed and reported with a significance level of *p* < 0.05. The confidence interval was 95%.

## Results

A total of 57 patients were screened, of whom 51 met the inclusion criteria and were included in the final analysis. Five patients were excluded due to insufficient tissue quality, and one was excluded due to non-standardized stump closure. Clinically relevant POPF occurred in 21 patients (41.2%), including 10 grade B and 11 grade C fistulas. There were no statistically significant differences between the POPF and No POPF groups regarding gender, age, underlying pathology or surgical approach (Table 1). Splenectomy was performed in 92.2% of patients. Patients in the POPF group had a significantly higher rate of severe postoperative complications (Clavien-Dindo grade ≥ III) compared to those in No POPF group (*p* < 0.0001). One 30-day mortality occurred in the POPF group. (Table 1)

**Table 1:** Patient characteristics and descriptive analysis

	POPF Group *n* = 21	No POPF Group *n* = 30	*p* value
Female sex, *n* (%)	5 (24)	13 (43)	0.232
Age ≥ 70, n (%)	9 (43)	12 (40)	> 0.99
Histopathology, n (%)			
PDAC	6 (29)	14 (47)	0.747
NET	6 (29)	6 (20)	
CP	2 (9)	2 (7)	
Metastasis	1 (5)	2 (7)	
Serous Cyst	1 (5)	2 (7)	
IPMN	2 (9)	0	
SPN	1 (5)	1 (3)	
Liposarcoma	1 (5)	1 (3)	
MCN	0	1 (3)	
Insulinoma	1 (5)	0	
Benign	0	1 (3)	
Wound Infection, n (%)	1 (5)	2 (7)	-
Splenectomy, n (%)	21 (100)	26 (87)	0.134
Clavien-Dindo Classification, n (%)			
0	0	17 (57)	*< 0.0001†*
I	0	3 (10)	
II	3 (14)	3 (10)	
III	11 (52)	6 (20)	
IV	6 (29)	1 (3)	
V	1 (5)	0	
30-day mortality, n (%)	1 (5)	0	-
Surgical approach, n (%)			
Open	20 (95)	26 (87)	0.782
Laparoscopic	1 (5)	3 (10)	
Robotic	0	1 (3)	

† Mann-Whitney U test (Clavien-Dindo ≥ III POPF vs. NoPOPF)

Quantitative immunohistochemical analysis was performed on tissue samples from the pancreatic resection margin to assess the relative area fractions of positive immunostaining for CD45 (leukocytes), CD68 (macrophages), CD31 (vascular endothelium), PanCK (exocrine cells), and adipocytes. These values were compared between the POPF and NoPOPF groups. (Table 2)

**Table 2:** Comparison the area of specifically stained cells and the area of adipocytes as a percentage of the total tissue area between two groups for each staining

	POPF Group *n* = 21	No POPF Group *n* = 30	T-value/ U-value	*p*
PanCK (X̄ ± SD)	0.4666 ± 0.196	t (49) = 0.4477	t(49)=0.4477	0.6563^†^
CD45	0.004136	0.002748	237	0.139^††^
CD68	0.001662	0.001148	262	0.3180^††^
CD31	0.02101	0.01782	277	0.4764^††^
Adipocytes-mean	0.04648	0.03757	270	0.3978^††^

† Unpaired t-test

†† Mann–Whitney U test

**Fig. 3:**
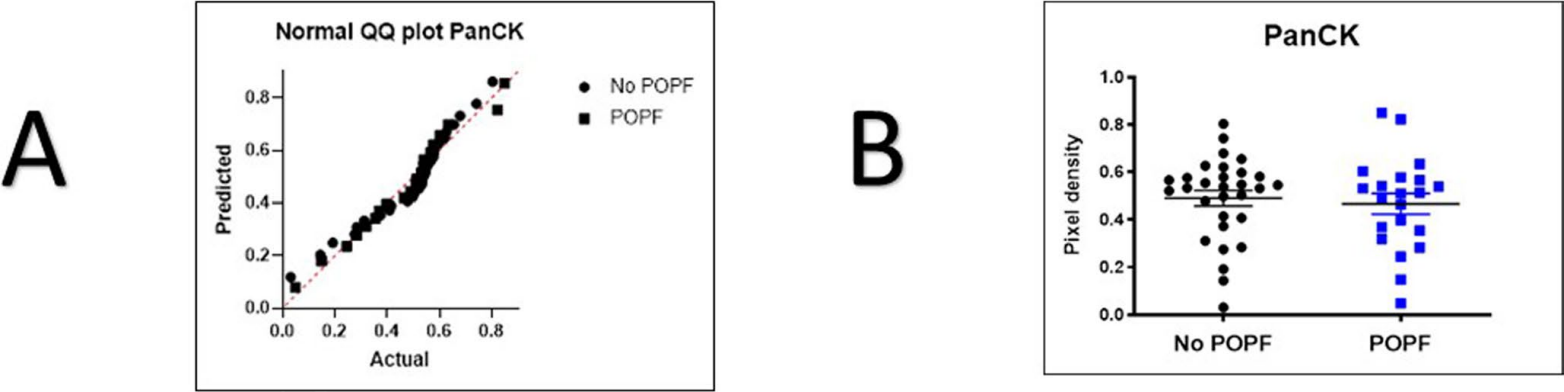
T-Test results for PanCK staining

PanCK staining, which represents the exocrine cell compartment, showed a mean relative area 0.4666 ± 0.196 in the POPF group and 0.49 ± 0.1747 in the NoPOPF group. Furthermore, the F-test demonstrated no significant difference in variances between the groups (*p* = 0.5601). The unpaired t-test yielded no statistically significant difference (t(49) = 0.4477, *p* = 0.6563) (Fig. 3).

**Fig. 4:**
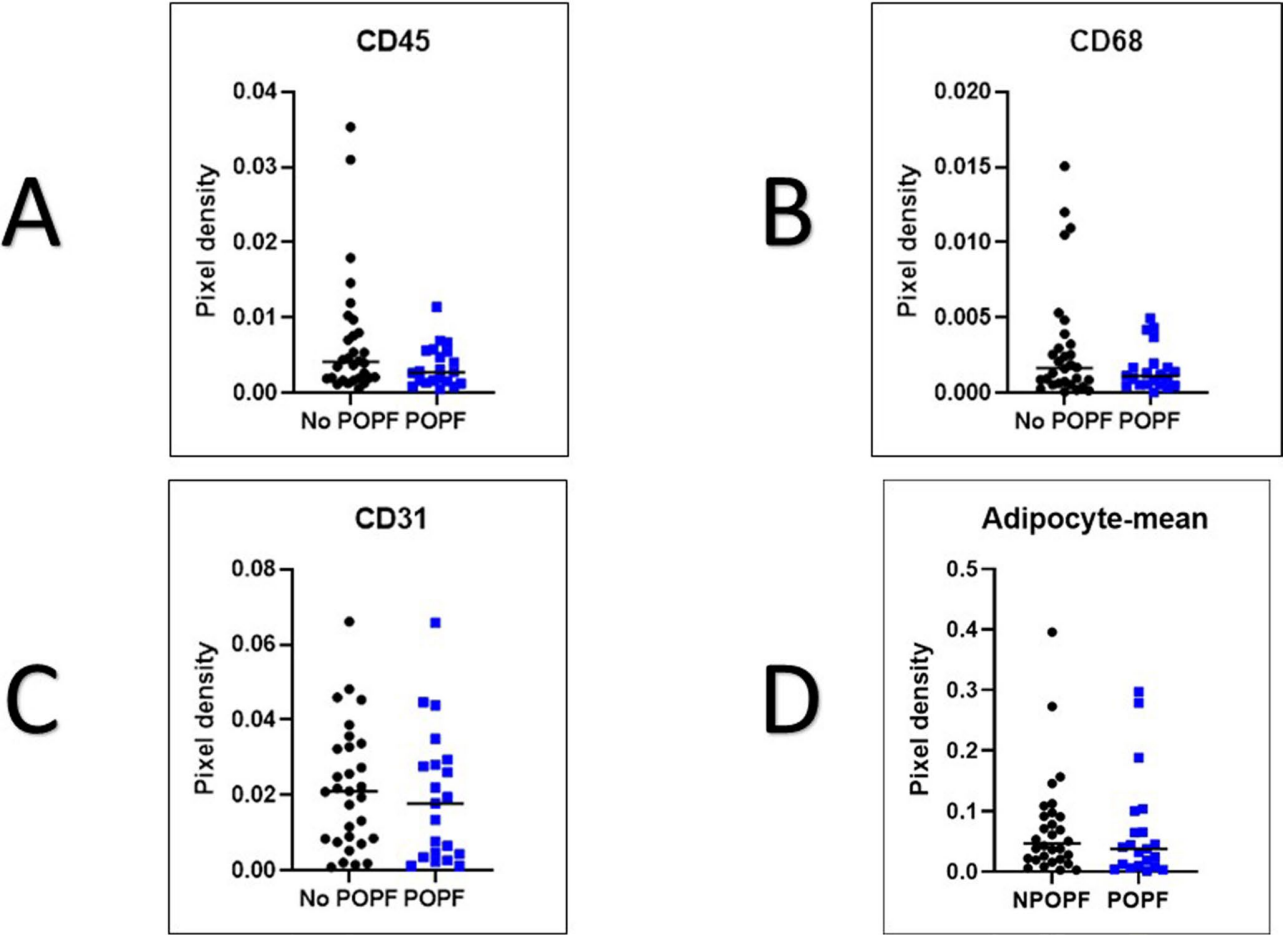
Scatter Plots comparing No POPF and POPF groups with mean bars considering Pixel Density **A**: CD45-positive cells **B**: CD68-positive cells **C**: CD31-positive cells **D**: Mean of Adipocyte-pixel density

Similarly, no significant differences were found in immune or stromal cell markers. The mean CD45-positive area was 0.002748 in POPF group and 0.004136 in NoPOPF group (*p* = 0.139). CD68-positive macrophage areas measured 0.001148 versus 0.001662 (*p* = 0.318) and CD31-positive endothelial areas were 0.01782 versus 0.02101 (*p* = 0.476) in the POPF and NoPOPF groups, respectively. Lastly, adipocyte content, as mesuared by unstained vacuolar cell area morphology, was similar between groups (POPF group = 0.03757 versus NoPOPF group = 0.04648; *p* = 0.398) (Fig. 4).

In summary, none of the evaluated histological parameters at the resection margin including immune cell infiltartions, exocrine cell content, vascular density, or adipocyte proportion showed a significant association with the development of POPF in this patient cohort.

## Discussion

In this study, we evaluated the histological composition of the pancreatic parenchymal resection margin in patients undergoing DP, focusing on inflammatory cell density (CD45, CD68), vascularization (CD31), exocrine tissue content (PanCK), and adipocyte percentage. Our aim was to determine whether these tissue characteristics were associated with the development of CR-POPF. Despite systematically applying quantitative, blinded immunohistochemical analysis, none of the investigated parameters demonstrated a statistically significant difference between patients who developed POPF and those who did not. Specifically, the proportions of leukocytes, macrophages, endothelial cells, exocrine cells, and adipocytes at the resection margin were comparable in both groups. These findings suggest that the cellular and stromal composition of the resection margin, as assessed by these markers, does not appear to play a decisive role in POPF formation after DP in this cohort.

The relatively high CR-POPF rate observed in this cohort likely reflects a selection bias of the rather small cohort of patients, who have most likely included cases with high risk pancreas (e.g. soft pancreas, obese patients). Comparable CR-POPF rates have been reported in recent DP series applying standardized ISGPS criteria [14–16]. 

Histopathological characteristics of the pancreas have long been recognized as key determinants of POPF risk, particularly following PD. Numerous studies in the PD setting have identified a “soft, acinar-rich gland with little fibrosis” as a prime risk factor for anastomotic leak. Mathur et al. first demonstrated that patients who developed POPF after PD had significantly increased pancreatic fat and reduced fibrosis and decreased vascular density on histology compared to those without fistula, leading to the conclusion that a “fatty pancreas” predisposes to fistula formation [17]. Pereira et al. reported that a hard, fibrotic pancreatic texture and dilated duct were associated with absence of POPF after PD, whereas a soft gland and small duct strongly predicted its occurrence [18]. Ridolfi et al. found that a low fibrosis/inflammation score at the resection stump was the main determinant of POPF after PD, underscoring the protective effect of desmoplastic (firm) pancreatic parenchyma [19]. Recent studies have focused on quantifying acinar cells at the pancreatic neck margin is an independent predictor of POPF following PD [20, 21]. Sugimoto et al., included 145 PD patients, a significant association was found between pancreatic parenchymal pattern characterized by “decreased lobules with significant fat infiltration” and CR-POPF. It was observed that in patients with a soft pancreas, concurrent processes of fatty infiltration and decreased lobules may progress together, potentially increasing the risk of POPF [22]. Soft pancreas corresponded with histologically lower acinar (lobular) fraction, higher fatty infiltration, small main duct diameter and thick parenchyma. These parameters were each independently associated with higher rates of clinically relevant POPF. In contrast, patients with inherently hard, fibrotic pancreases due to chronic pancreatitis or tumors causing desmoplasia rarely developed fistulas. Consistently, Tanaka et al. observed that none of the patients with a histologically “hard” pancreas in their series experienced a leak [23]. Laaninen et al. and Teränen et al. demonstrated that high acinar cell content (> 40%) at the resection margin predicts postoperative complications and clinically relevant POPF after PD [24, 25]. Taken together, these studies established that limited fibrosis and abundant acinar cells in the remnant pancreas are strongly linked to heightened POPF susceptibility after PD. The biological rationale is clear: residual acinar-rich, poorly fibrotic pancreatic tissue continues to secrete proteolytic enzymes into a freshly constructed anastomosis, creating a fragile interface vulnerable to enzymatic disruption.

While gland texture and histology are well-established predictors of POPF after PD, their relevance in DP remains controversial. Nahm et al. reported a 20.4% clinically relevant POPF rate after DP versus 11.2% after PD. They attributed this nearly two-fold increase to differences in pancreatic histology between the two operations: the resection margins of DP specimens had significantly higher acinar cell density and less fibrosis on average than those of PD [26]. Generally softer, enzyme-rich pancreatic tail inherently carries greater leak risk than the often-fibrotic pancreatic head. However, the findings of Halle-Smith et al. and Eshmuminov et al., who both reported that histological factors such as fibrosis, fat content, and duct size showed no significant association with POPF in DP, despite confirming their predictive value in PD [7, 27]. These conflicting results highlight the unresolved role of pancreatic tissue composition in fistula formation after DP. The key anatomical and physiological difference is that, in DP, there is no pancreatic-enteric anastomosis; the pancreatic stump is simply sealed, often with a stapler or suture. This eliminates the mucosal healing interface that is central to anastomotic failure in PD. Consequently, the pathophysiology of POPF after DP may be less influenced by enzyme-secreting cellular components and more by mechanical and technical factors.

Although vascularization, tissue inflammation, fibrosis, adipocyte or exocrine cell percentage at the parenchymal resection margin of the pancreatic tissue appear to be histologically associated in the literature for PD, both histological and as a novel concept to evaluate pancreatic resection margin immunohistochemical methods are needed for DP. Therefore, our study, being specific to DP and including immunohistochemical analyses, will provide a new perspective to the literature. While the main factor contributing to the development of POPF in PD patients is pancreaticojejunostomy leakage, the etiology of POPF following distal pancreatectomy (DP) is more complex and multifactorial. Proposed mechanisms include mechanical failure of the transection line, particularly in patients with a soft, adipose-rich pancreas, in whom reduced parenchymal firmness and altered tissue integrity may impair staple-line security and promote enzymatic leakage; soft pancreatic texture has been consistently identified as an independent risk factor for POPF in DP cohorts (e.g., soft pancreas texture associated with higher fistula rates) [28]. Obesity and increased visceral adiposity have also been implicated, with higher body mass index shown to correlate with elevated POPF risk after DP, potentially reflecting excess fat within and around the gland contributing to inflammation and impaired local healing [29, 30]. In addition, intraductal hypertension within a blind-ended pancreatic stump — possibly exacerbated by high exocrine output or functional outflow disturbances due to Sphincter of Oddi dysfunction [31] — has been proposed as a contributor to transection-line disruption and biochemical leak formation, consistent with conceptual models of stump pressure dynamics and postoperative leakage propensity. Together, these hypotheses underscore the interplay between mechanical, metabolic, and biological factors in the development of POPF after DP.

In addition to the precise assessment of POPF risk, the implementation of safer surgical techniques and targeted perioperative management strategies is necessary, particularly for high-risk patients such as those with fatty pancreas. Given that pancreatic steatosis is associated with increased gland fragility and impaired anastomotic healing, optimization strategies may need to address not only technical factors but also the biological characteristics of the pancreatic remnant. Various stump closure techniques, pharmacological interventions, and reinforcement methods have been explored, yet no consensus has been reached on a standardized prevention strategy [32–34]. In this context, perioperative interventions aimed at modulating the inflammatory response have gained attention. Prophylactic corticosteroids, as demonstrated by Antila et al., may help mitigate postoperative inflammation and reduce the clinical impact of leaks, regardless of the underlying gland texture [35]. Whether patients with fatty pancreas represent a subgroup that could particularly benefit from such biologically targeted perioperative approaches warrants further investigation.

This study has several limitations. First, its retrospective, single-center design limits causal inference and generalizability. Second, the pilot sample size restricts statistical power and precludes multivariable risk modeling, including stratification by established fistula risk scores. Third, although surgical technique was standardized, subtle technical nuances such as stapler selection, reinforcement materials, or duct ligation strategies were not analyzed in detail. Finally, patient-related biochemical and functional parameters were not incorporated. These limitations underline the need for larger, prospective, multicenter studies integrating clinical, technical, and histological variables.

Our findings challenge the routine assumption that pancreatic tissue composition predicts POPF after DP. Despite the established role of acinar cell predominance and low fibrosis as risk factors for POPF after PD, our findings—consistent with several previous reports—demonstrate no significant association between these histological features and POPF in the setting of DP. While we are continuing to expand our dataset with additional patient samples to strengthen these observations, the current results reinforce the emerging view that, unlike in PD, tissue composition alone is not a reliable predictor of POPF after DP, and that technical, mechanical, and systemic factors likely play a more dominant role in fistula development in this context.

Future research should focus on multimodal risk stratification strategies, combining clinical, operative, histological, and functional tissue assessments to better predict and prevent POPF after DP. Larger, prospective, multicenter studies are needed to validate these findings and to define the most effective prevention and management strategies for this common and impactful complication.

## Data Availability

All data are available from the Authors upon reasonable request.
